# Occupational Health Physicians’ Perspectives on Factors Influencing Return-to-Work Decisions for Employees With Mental Health Disorders: A Retrospective Observational Study

**DOI:** 10.7759/cureus.86189

**Published:** 2025-06-17

**Authors:** Shotaro Doki, Daisuke Hori, Mami Ishitsuka, Asako Matsuura, Hotaka Tsukada, Wakako Migaki, Norishige Kanai, Reem Al Assaad, Shin-ichiro Sasahara

**Affiliations:** 1 Occupational Psychiatry and Aerospace Medicine, Institute of Medicine, University of Tsukuba, Tsukuba, JPN; 2 Graduate School of Comprehensive Human Sciences, University of Tsukuba, Tsukuba, JPN; 3 School of Nursing at Narita Department of nursing, International University of Health and Welfare, Narita, JPN; 4 Division of Translational Nursing, Toho University, Funabashi, JPN; 5 Faculty of Medicine, Department of Clinical Psychology, Kagawa University, Takamatsu, JPN; 6 Research and Development Center for Lifestyle Innovation, University of Tsukuba, Tsukuba, JPN

**Keywords:** fitness for work, occupational mental health, return-to-work, sick leave, worker

## Abstract

Introduction

Assessing fitness for work among employees with mental health disorders presents significant challenges. While it is fundamentally assumed that employees must be capable of adequately fulfilling their job responsibilities, occupational health professionals frequently encounter difficulties in making such determinations. This study aims to elucidate the factors considered by occupational physicians, particularly those specializing in psychiatry when evaluating the work eligibility of employees experiencing mental health issues.

Methods

We analyzed a subset of occupational physician interview records collected over a 14.5-year period at a higher education institution. A total of 1,381 interviews involving 184 individuals were included. For two groups, employees currently working and those on leave, we used the occupational physician’s decision as the dependent variable. A generalized linear mixed model (GLMM) was employed to estimate the odds ratios (ORs) of factors associated with the physician’s decision to recommend sick leave versus continued work without leave.

Results

Among employees currently working, factors associated with the occupational physician’s decision to recommend sick leave rather than continued work included other occupations (OR [95% CI] = 5.43 [1.16, 25.42]), reduced sense of sleep quality (OR [95% CI] = 4.22 [1.07, 16.67]), and loss of appetite (OR [95% CI] = 6.81 [1.36, 34.2]). Among employees on sick leave, the only factor associated with a return-to-work decision, as opposed to continued sick leave, was the total duration of prior sick leave (in months) (OR [95% CI] = 0.94 [0.91, 0.96]).

Conclusions

The present study revealed that occupational physicians specializing in psychiatry place significant emphasis on lifestyle factors such as sleep and appetite when assessing fitness for work. When occupational physicians assessed fitness to return to work for employees on sick leave, they focused on the duration of the leave, which could serve as a potential risk factor for relapse.

## Introduction

Mental health issues among workers have become a significant concern in occupational health. According to the 2022 Survey on Industrial Safety and Health conducted by the Ministry of Health, Labour, and Welfare of Japan, 82.2% of workers reported experiencing strong anxiety, worry, or stress related to work. Additionally, 13.3% of workplaces had employees who had taken a leave of absence for more than one month or had resigned due to mental health problems within the past year [1]. Despite this, only 63.4% of workplaces reported implementing measures to address mental health, indicating that the response to mental health issues remains an urgent challenge in many work environments [1].

Determining whether employees with mental health problems are fit to continue working, require a leave of absence, or are ready to return to work following a sick leave is a routine and important responsibility for healthcare professionals [2]. Although previous studies have examined whether adherence to guidelines can facilitate an earlier return to work for employees with common mental disorders, the effectiveness of such adherence remains inconclusive [3].

Mental health disorders are often less visible than physical illnesses, with symptoms that may be transient and that are frequently reliant on self-reported information, making fitness-for-work decisions substantially more complex [4]. While the fundamental assumption is that employees must be able to provide sufficient labor to their employers, a variety of factors, such as fluctuations in psychiatric symptoms, the risk of relapse, the compatibility between the worker’s condition and job duties, and the worker’s own insight into their condition, often interact in complex ways, posing significant challenges for occupational health professionals [4].

Against this background, the Ministry of Health, Labour, and Welfare issued the Guidelines for Supporting the Return to Work of Employees Who Have Taken Leave Due to Mental Health Problems in 2009, emphasizing the importance of collaboration among attending physicians, occupational physicians, and employers in supporting employees' return to work [5]. However, it remains unclear which factors influence the actual decision-making processes of occupational physicians when determining fitness for work. Unlike clinical physicians, occupational physicians also provide recommendations to employers and workplace supervisors, resulting in a mutual influence between their decisions and the work environment. Furthermore, since risk perception differs depending on workers’ experience and gender, there is no standardized approach to decision-making among occupational physicians [6]. In particular, the decision-making processes of occupational physicians specializing in psychiatry often involve subjective elements, making standardization difficult and resulting in frequent reliance on individual clinical experience [6].

This study aimed to identify the factors considered by occupational physicians specializing in psychiatry when making fitness-for-work decisions, based on long-term records of occupational health interviews conducted at a higher education institution. For workers, taking a leave of absence or returning to work is a critically important event, both in terms of their career and financial well-being. However, decisions made by occupational physicians have often been highly individualized, lacking consistency and standardized assessment criteria. By clarifying these decision-making factors, the study seeks to contribute to the visualization and potential standardization of the assessment process, ultimately supporting improvements in the quality of future occupational health practices.

A part of this article was previously presented as a meeting abstract at the Japan Society for Occupational Health Annual Scientific Meeting on May 25, 2024.

## Materials and methods

This study was a retrospective observational study based on records of occupational physician interviews conducted over a 14.5-year period, from April 2004 to the end of September 2018, at a higher education institution. The study population consisted of employees who underwent interviews due to mental health-related issues. From these records, only interviews that included documented fitness-for-work decisions by the occupational physician were extracted for analysis.

**Figure 1:**
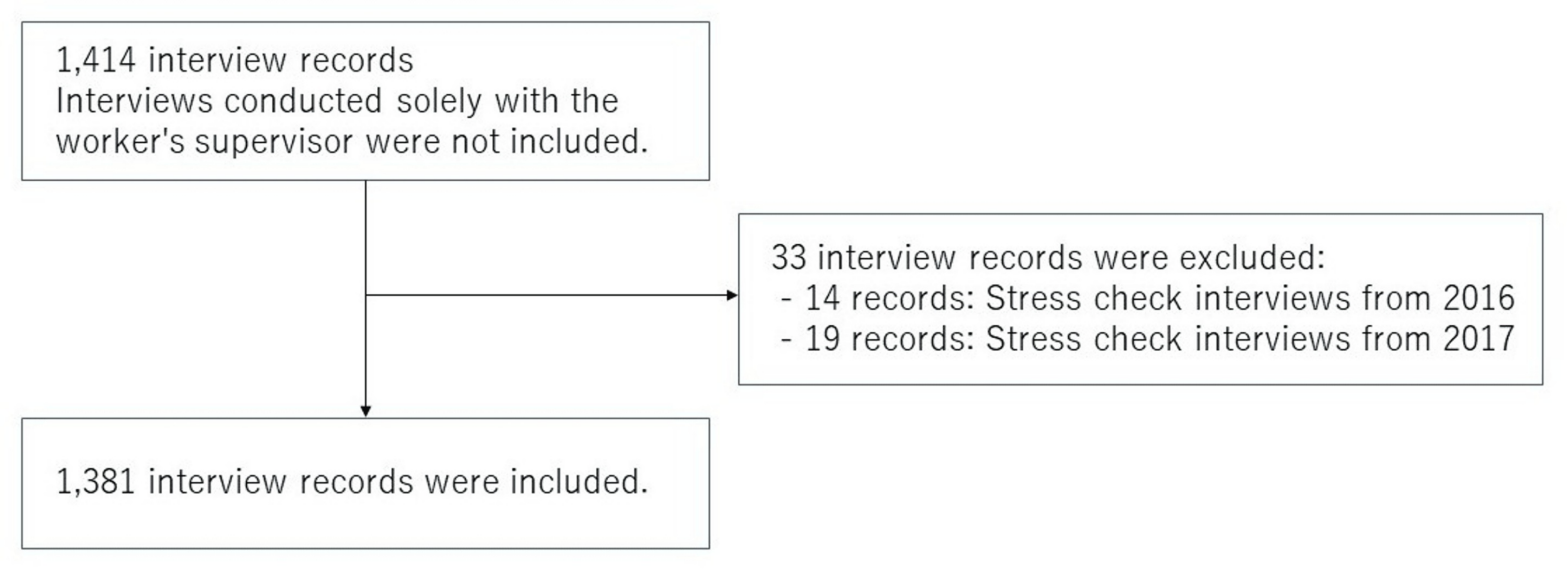
Flow diagram of data inclusion and exclusion

As all interview records were handwritten, the research team transcribed them verbatim and digitized the data. The dataset does not include consultations conducted solely with the worker’s supervisor. Occupational health interviews related to the stress check screening system, which was mandated by law in December 2015 (14 interviews in 2016 and 19 interviews in 2017), were excluded from the dataset (Figure 1). Since occupational health interviews are semi-formal in nature, there were no missing data such as unidentified names. A total of 1,381 interviews involving 184 employees were included in the analysis. Eight occupational physicians conducted interviews. Each employee’s work status was labeled as either “currently working” or “on sick leave.” For employees currently working, the occupational physician’s judgment was categorized as either “continued work” or “sick leave required.” For employees on sick leave, judgments were categorized as either “continued sick leave” or “return to work.”

Statistical analysis

Missing values for age were imputed using multiple imputations. Of the 1,381 interview records, 397 cases (28.7%) required imputation. The dependent variable was defined as either “continued work” or “sick leave required” for employees currently working and as “continued sick leave” or “return to work” for employees on leave. Odds ratios (ORs) for factors associated with each judgment were calculated using generalized linear mixed models (GLMM). To account for repeated measures within individuals, employee ID was specified as the subject variable in the model.

Independent variables included age, sex, occupational category, sleep-related indicators (difficulty falling asleep, nighttime awakening, early morning awakening, sense of sleep quality), appetite status, interpersonal relationship problems, presence or absence of psychiatric prescriptions, and total accumulated duration of sick leave. Each variable was defined and categorized based on descriptions in the interview records. The format for coding each variable is provided in the Appendices.

Statistical analyses were performed using IBM Corp. Released 2022. IBM SPSS Statistics for Windows, Version 30. Armonk, NY: IBM Corp. As multiple imputation and GLMM cannot be performed simultaneously in SPSS, one imputed dataset from the multiple imputation process was used to conduct the GLMM analysis. A significance level of 0.05 was adopted, and 95% confidence intervals (CIs) were calculated. This study was approved by the Ethics Committee of the Institute of Medicine, University of Tsukuba (Approval No. 1326).

## Results

Table 1 presents the basic characteristics of the participants. The mean age was 40.3 years (standard deviation 11.7), with 89 males (48.4%) and 95 females (51.6%). The most common occupational category was administrative staff, accounting for 98 individuals. The most frequent entry for educational background was “unknown” (not recorded), with 120 individuals. Among the interview records that included a documented diagnosis, the most common was depression (29 cases), followed by adjustment disorder (13 cases); however, 106 records did not include any diagnosis (Table 2). The total number of interviews was 970 for employees currently working and 408 for those on sick leave.

**Table 1:** The basic characteristics of the participants SD: standard deviation

	N (%)	mean (SD)
Age, years	-	40.3 (11.7)
Sex		
Male	89 (48.4)	-
Female	95 (51.6)	-
Occupation		
Faculty	25 (13.6)	-
Administrative	98 (53.3)	-
Specialist	9 (4.9)	-
Others	52 (28.2)	-
Number of sick leaves	-	0.4 (0.6)

**Table 2:** Documented diagnosis Multiple diagnoses were allowed.

Diagnosis	N (%)
No diagnosis recorded	106 (57.6)
Depression	29 (15.8)
Adjustment disorder	13 (7.1)
Autonomic disorder	5 (2.7)
Anxiety	5 (2.7)
ADHD	2 (0.11)
Sleep disorder	1 (0.5)
Autism	1 (0.5)
Schizophrenia	1 (0.5)
Somatic symptom disorder	1 (0.5)
Bipolar disorder	0 (0.0)
Others	24 (13.0)

Among employees currently working, factors associated with the occupational physician’s decision to recommend sick leave rather than continued work included other occupations (OR [95% CI] = 5.43 [1.16, 25.42]), absence of sense of sleep quality (OR [95% CI] = 4.22 [1.07, 16.67]), and loss of appetite (OR [95% CI] = 6.81 [1.36, 34.2]) (Table 3). For employees on sick leave, the only factor associated with a return-to-work decision as opposed to continued sick leave was the total duration of prior sick leave (in months) (OR [95% CI] = 0.94 [0.91, 0.96]) (Table 4).

**Table 3:** Odds ratios for factors associated with the occupational physician’s decision to recommend sick leave versus continued work among employees currently working CI: confidence interval Binary logistic regression with a generalized linear mixed model

	Odds	95% CI
Age, years	0.969	[0.908, 1.034]
Sex, male	0.768	[0.195, 3.026]
Occupation		
Administrative	reference	
Technician	0.835	[0.049, 14.112]
Faculty	3.282	[0.541, 19.908]
Others	5.427	[1.159, 25.419]
Difficulty falling asleep	0.763	[0.092, 6.352]
Nocturnal awakening	0.396	[0.065, 2.413]
Early morning awakening	2.764	[0.277, 27.599]
Loss of the sense of sleep quality	4.223	[1.070, 16.665]
Appetite		
Loss	6.810	[1.355, 34.221]
Normal	reference	
Increased	<0.001	[0.000, -
Interpersonal problems	0.758	[0.125, 4.610]
Medication	0.995	[0.274, 3.613]
Total duration of sick leave, months	0.889	[0.703, 1.124]

**Table 4:** Odds ratios for factors associated with the occupational physician’s decision to recommend return to work versus continued sick leave among employees on leave CI: confidence interval Binary logistic regression with a generalized linear mixed model

	Odds	95% CI
Age, years	1.016	[0.986, 1.047]
Sex, male	1.470	[0.670, 3.225]
Occupation		
Administrative	reference	
Technician	1.320	[0.535, 3.254]
Faculty	0.741	[0.220, 2.495]
Others	0.735	[0.287, 1.878]
Difficulty falling asleep	0.835	[0.137, 5.098]
Nocturnal awakening	2.270	[0.783, 6.577]
Early morning awakening	0.310	[0.021, 4.618]
Loss of the sense of sleep quality	0.403	[0.101, 1.608]
Appetite		
Loss	0.202	[0.015, 2.719]
Normal	reference	
Increased	<0.001	[0.000, -]
Interpersonal problems	0.719	[0.202, 2.558]
Medication	0.709	[0.314, 1.599]
Total duration of sick leave, months	0.935	[0.906, 0.964]

## Discussion

This study examined the factors considered by occupational physicians specializing in psychiatry when determining the fitness for work of employees. The results indicated that, for employees currently working, decisions to recommend sick leave were associated with fundamental lifestyle-related factors such as sleep and appetite. In contrast, for employees on leave, the only factor significantly associated with a return-to-work decision was the total duration of prior sick leave. These findings suggest that occupational physicians assess not only the employee’s subjective complaints but also the overall organization of daily life and past history in a comprehensive manner when evaluating mental stability. The insights gained from this study may serve as practical guidance for occupational physicians and occupational health professionals when making decisions regarding work continuation or sick leave in the future.

Many of the employees categorized as having “other occupations” at the higher education institution surveyed in this study were nurses. In Japan, nurses are known to experience high levels of psychological stress and have a relatively high incidence of taking sick leave [7]. Unlike those in other occupational groups, nurses often seek consultation with occupational physicians only after their conditions have significantly deteriorated. This suggests that early intervention is more difficult in the case of nurses, which may explain the strong tendency toward decisions of “sick leave required.” Shift work among nurses has also been reported to be associated with increased depressive symptoms and a higher prevalence of sleep disturbance [8,9].

Among the sleep-related factors, the sense of sleep quality was associated with the decision to recommend sick leave. Previous studies have reported that the amount of sleep required varies widely among individuals [10] and that subjective sleep quality has a greater impact on depressive mood than objective sleep duration [11]. Changes in appetite were also identified as an important factor, as appetite loss is known to be one of the core symptoms of depression [12]. These findings suggest that occupational physicians take such physical symptoms into account when evaluating whether an employee is fit to continue working.

On the other hand, in decisions regarding the return to work of employees on sick leave, occupational physicians tended to be more cautious when assessing workers with longer durations of leave. This cautious approach is likely due to reports indicating that longer sick leave is associated with a higher risk of relapse [13], suggesting that physicians prioritize long-term employment stability in their evaluations. Some employees may wish to return to work before they have fully recovered, due to concerns about job security or financial reasons. However, repeated relapses significantly increase the risk of requiring further sick leave [14], indicating that premature return to work should be avoided. When occupational physicians consider an employee’s return to work, they tend to focus on the current symptoms. This may explain why diagnostic information was largely missing in the results presented in this study.

This study has several limitations. First, it did not incorporate information from the attending physicians’ medical certificates and number of sick leaves, which may have influenced the results if considered. Decisions made by occupational physicians do not always accurately reflect the worker’s medical condition or lead to the most appropriate course of action. Second, as all records were handwritten, there is a possibility of misclassification due to discrepancies in the timing of information transmission from workers to occupational physicians and the timing of digitization of the records. Caution is also needed regarding the generalizability of the findings, as the study was conducted at a single institution. Furthermore, the possibility of a healthy worker effect exists, as data from healthy employees who did not consult with an occupational physician were not included. Additionally, important factors such as the presence of residual symptoms, history of participation in return-to-work (rework) programs, engagement in commuting training (e.g., visiting a library), the attending occupational physician, and the degree of workplace readiness to accommodate returning employees were not available in the dataset. These variables could potentially act as unmeasured confounding factors. Future research should aim to collect and analyze more detailed data to further clarify the factors occupational physicians consider when making decisions regarding an employee’s return to work or continued sick leave.

## Conclusions

This study revealed that occupational physicians specializing in psychiatry place particular emphasis on lifestyle factors such as sleep and appetite when assessing fitness for work. In particular, the sense of sleep quality was found to influence decisions to recommend sick leave, suggesting that subjective sleep quality is an important indicator in evaluating mental health status. Furthermore, in return-to-work decisions, the total duration of prior sick leave emerged as a key factor in the occupational physicians’ assessments.

## Appendices

Symptom coding for all variables

Sleep: 00:00 ~ 00:00/Total: 00.00 hours

Difficulty falling asleep: Absent/Present

Middle-of-the-night awakening: Absent/Present

Early morning awakening: Absent/Present

Sense of sleep quality (feeling of restful sleep): Absent/Present

Daytime sleepiness: Absent/Present

Napping: Absent/Present; Duration: 00 hours/day

Appetite: Good or Average/Poor or Decreased/Increased or Excessive

Weight: 00 kg; Change: Increased by 00 kg/Decreased by 00 kg

Facial Expression

Mood: Euthymic (normal, good)/Depressed (bad)/Elated, manic, hypomanic

Motivation: Absent/Present

Concentration: Absent/Present

Fatigue or tiredness: Absent/Present

Anxiety: Absent/Present

Suicidal thoughts: Absent/Present

Somatic Symptom

Palpitations: Absent/Present

Headache: Absent/Present

Back pain: Absent/Present

Abdominal pain: Absent/Present

Diarrhea: Absent/Present

Alcohol consumption: Absent/Present

If present: Type (beer, shochu, wine, sake, sour, etc.) - 000 ml/day, 0 days/week

Smoking: Absent/Present If present: 00 cigarettes/day

Exercise: Absent/Present If present: Type of exercise:

Duration: 00 minutes/session, Frequency: 00 times/week

How weekends are spent:

Diagnosis:

Hobbies:

Medications: Psychiatric: Absent/Present Physical: Absent/Present

Past medical history (PH): Psychiatric: Absent/Present Physical: Absent/Present

Commute: Duration: 00 minutes Means: Car/Bicycle/Train/Bus/Walking

Free-text field:
